# Time of day of exercise does not affect the beneficial effect of exercise on bone structure in older female rats

**DOI:** 10.3389/fphys.2023.1142057

**Published:** 2023-10-26

**Authors:** Jay J. Cao, Brian R. Gregoire

**Affiliations:** USDA, Agricultural Research Service, Grand Forks Human Nutrition Research Center, Grand Forks, ND, United States

**Keywords:** exercise, circadian rhythm, bone structure, time of day, insulin-like growth factor

## Abstract

**Background:** Circadian clock genes are expressed in bone and biomarkers of bone resorption and formation exhibit diurnal patterns in animals and humans. Disruption of the diurnal rhythms may affect the balance of bone turnover and compromise the beneficial effects of exercise on bone.

**Objective:** This study investigated whether the time of day of exercise alters bone metabolism in a rodent model. We hypothesized that exercise during the active phase results in greater bone mass than exercise during the rest phase in older female rats.

**Methods:** Fifty-five, female 12-month-old Sprague Dawley rats were randomly assigned to four treatment groups (*n* = 13–14/group). Rats were subjected to no exercise or 2 h of involuntary exercise at 9 m/min and 5 days/wk for 15 weeks using motor-driven running wheels at Zeitgeber time (ZT) 4–6 (rest phase), 12–14 (early active phase), or 22–24 (late active phase). ZT 0 is defined as light on, the start of the rest phase. A red lamp was used at minimal intensity during the active, dark phase exercise period, i.e., ZT 12–14 and 22–24. Bone structure, body composition, and bone-related cytokines in serum and gene expression in bone were measured. Data were analyzed using one-way ANOVA followed by Tukey-Kramer *post hoc* contrasts.

**Results:** Exercise at different ZT did not affect body weight, fat mass, lean mass, the serum bone biomarkers, bone structural or mechanical parameters, or expression of circadian genes. Exercise pooled exercise data from different ZT were compared to the No-Exercise data (*a priori* contrast) increased serum IGF-1 and irisin concentrations, compared to No-Exercise. Exercise increased tibial bone volume/total volume (*p* = 0.01), connectivity density (*p* = 0.04), and decreased structural model index (*p* = 0.02). Exercise did not affect expression of circadian genes.

**Conclusion:** These data indicate that exercise is beneficial to bone structure and that the time of day of exercise does not alter the beneficial effect of exercise on bone in older female rats.

## Introduction

Many biochemical processes and physiological functions of mammals, such as sleep/wake, fast/feeding, body temperature, hormone secretion, and locomotor activity, display biological or circadian rhythms. These rhythms are controlled by circadian clocks with master clock located in the suprachiasmatic nucleus of the hypothalamus and peripheral clocks in the almost every tissue and organ system including muscle and bone (Aoyama and Shibata, 2017). At the molecular level, both central and peripheral clocks are regulated by the transcriptional and translational feedback loops of core clock genes (Aoyama and Shibata, 2017). Disruptions of biological rhythms, such as the synchrony with the natural daily light-dark cycle or timing of fast/feeding, contribute to many health disorders in humans such cognitive impairments, mood disturbances, increased risk of cardiometabolic disorders, obesity (Zee et al., 2013; Fonken and Nelson, 2014), or increased risk of osteoporosis and bone fractures (Amstrup et al., 2013; Swanson et al., 2017a).

Clock genes regulate bone homeostasis since they are expressed in bone cells (Samsa et al., 2016; Aoyama and Shibata, 2017; Takarada et al., 2017; Yuan et al., 2017) and about one-third of genes expressed in mouse calvarial bone show a rhythmic expression (Zvonic et al., 2007). Skeletal muscle has an extensive network of clock-controlled genes and physical strength and muscle mitochondrial function peak in the late afternoon (Gabriel and Zierath, 2019). Studies have demonstrated that bone cell functions and bone mass may be directly or indirectly regulated by circadian genes. In mice, disruption of circadian genes affects functions and activities of osteoblasts or osteoclasts and bone mass. Mice with global or osteoblast-specific deletion of Bmal1 have a low bone mass due to increased bone resorption (Takarada et al., 2017) and osteoclast-specific Bmal1 knockout mice have a high bone mass phenotype due to reduced osteoclast differentiation (Xu et al., 2016).

Bone is a dynamic tissue that undergoes significant turnover, a process called bone modeling and remodeling, throughout the lifespan and bone mass is regulated between bone resorption by osteoclasts and bone formation by osteoblasts. In animal models, disruption of the balance of bone resorption and formation can lead to osteoporosis (Lacey et al., 1998; Mundy, 2007). Bone metabolism regulating biomarkers, such as bone resorption marker, C-terminal telopeptide of type 1 collagen (CTX) and bone formation marker, osteocalcin, exhibit distinct diurnal oscillations in humans and animals, that may affect rates of bone formation and resorption (Srivastava et al., 2001; Shao et al., 2003; Dudek and Meng, 2014; Redmond et al., 2016; Swanson C. et al., 2017; Swanson et al., 2017c). Bone resorption and formation markers exhibit diurnal patterns with light/dark cycle in nocturnal rats (Shao et al., 2003) that peaked during the light period when animals are in rest phase and the patterns are opposite to those observed in humans according to clock time (Bjarnason et al., 2002; Redmond et al., 2016; Swanson C. et al., 2017; Swanson et al., 2017c).

The benefit of exercise (Ex) on bone mass and strength is well-known (King et al., 2009). Exercise stimulates osteoblast proliferation and differentiation and reduces apoptosis of osteoblasts and osteocytes, the latter are sensors for mechanical loading. At the molecular level, the process is regulated through WNT and β-catenin signaling pathways (Ehrlich and Lanyon, 2002; Sawakami et al., 2006; Bonewald and Johnson, 2008).

Timing of exercise affects many physiological responses (Schroeder et al., 2012; Schroder and Esser, 2013; Sasaki et al., 2014; Iwayama et al., 2015; Peek et al., 2017), e.g., altering the time of exercise alters circadian genes in muscle and heart and affects physiology in mice (Schroder and Esser, 2013) and wheel running at the end, not the beginning, of the active phase, results in greater improvement of rhythmic deficits in vasoactive intestinal polypeptide-deficient mice (Schroeder et al., 2012). Bouchard et al. (2022) demonstrated that mechanical loading of tibia at early active phase (Zeitgeber time, ZT14) increased endocortical bone formation and decreased expression of Sost and Dkk1, Wnt-signaling inhibitors, in young mouse tibia, compared to loading at resting phase (ZT2). Exercise can reset the molecular circadian clock and ameliorate the negative effects of disrupted sleep patterns (Gabriel and Zierath, 2019). Optimizing the timing of exercise has been proposed as one of the therapeutic interventions for preventing metabolic diseases.

Whether the time of day of exercise alters the beneficial effects of exercise on bone has not yet been investigated. Therefore, we conducted an animal study and evaluated the impact of involuntary running at the rest phase, early or late active phase on bone biomarkers and bone structure in 12-month-old female rats, as rats at this age have reached peak bone mass and bone loss occur. Previously, Yeh J.K et al. demonstrated that 16 weeks treadmill exercise increased bone density in tibia and vertebra in female rats aged 14 months (Yeh et al., 1993). We hypothesized that exercise during the active phase results in greater bone mass than exercise during the rest phase in older female rats.

## Methods and materials

### Animals, diet, and treatments

Fifty-five female Sprague Dawley rats, 12-month-old, were obtained from Envigo (Cumberland, VA). Twelve-mo-old female rats were used as a model for age-related bone loss as rats at this age have reached peak bone mass (Wronski et al., 1989). We expected similar results in male rats in response to the timing of exercise, although the degree of response to exercise may be different between male and female animals. The animal protocol for the study was reviewed and approved by the United States Department of Agriculture, Agricultural Research Service, Grand Forks Human Nutrition Research Center Institutional Animal Care and Use Committee. Animals were maintained and processed in accordance with the NIH Guide for the Care and Use of Laboratory Animals. Rats were individually housed in double sized suspended stainless-steel cages located in an environmentally controlled pathogen-free facility. To minimize the stress to animals, environmental enrichment comprising of an empty glass food cup and/or bio tunnels and rodent nesting sheets were provided. Rats were allowed to acclimate to our animal facility for 1 wk before being assigned to the experimental treatments. Rats had free access to the same diet (Research Diet D10012M, based on AIN-93M mature rodent diet, Research Diets Inc., New Brunswick, NJ) and deionized water throughout the study. Food intake was measured on 2 consecutive days every other week.

**FIGURE 1:**
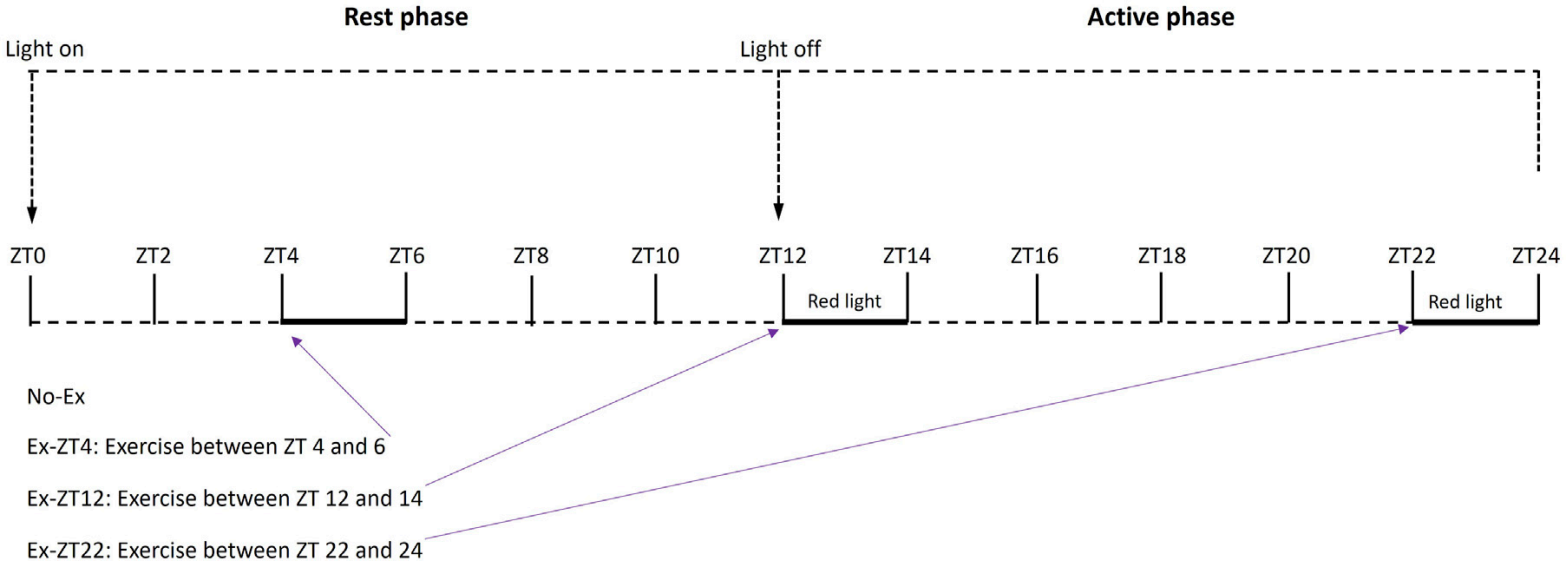
Schematic of treatment protocol of rats exercised (Ex) during different Zeitgeber time (ZT).

Then, rats were randomly assigned by body weight to 4 treatment groups as follows. Rats were subjected to no exercise (No-Ex, *n* = 14) or 2 h of involuntary exercise at ZT between 4–6 (Ex-ZT4, rest phase, *n* = 13), 12–14 (Ex-ZT12, early active phase, *n* = 14), or 22–24 (Ex-ZT22, late active phase, *n* = 14), as described in Figure 1. Rats were maintained with a standard automatically-controlled 12-h light/12-h dark cycle. Light was on at ZT0 as the start of the rest phase and the light was off at ZT12 as the start of the active phase for rats. Rats were housed in three separate rooms: ZT12, ZT22, and No-Ex and Ex-ZT4 in the same room. The on and off light cycles for each room were adjusted individually to allow us to perform exercise, animal care, and other experimental procedures during the daytime (natural clock time) for either ZT4, ZT12, or ZT22. Lighting intensity was the same for all three animal rooms. Rate were subjected to forced exercise on motor driven running wheels (Lafayette Instruments Co., Lafayette, IN) for a total of 15 weeks. To prevent injury, the exercise regimen started at the speed of 4 m/min for 1 h/d, 5 days/wk and gradually progressed to 9 m/min for 2 h/d over a 5-week period. Then, rats were maintained at this level of activity for a 10-week period. The exercise protocol was considered moderate exercise and was developed and optimized based on previous work at our research center (Cao et al., 2015; Picklo and Thyfault, 2015; Cao, 2018) and other studies with rats (Uysal et al., 2017; Quiroga et al., 2020). Exercise was performed in an adjacent separate room equipped with regular fluorescent and red ceiling lamp (GE F32T8/R/24/ECO/CVG9) that were turned on and off depending on the exercise timing.

### Sample collection and preparation

Rats were euthanized with intraperitoneal injection of a cocktail of ketamine:xylazine (Animal Health Co. and Phoenix Scientific, respectively) at 58 and 8.4 mg/kg body weight for ketamine and xylazine, respectively. To minimize the effect of timing of euthanasia on endpoint measurements, animals were sacrificed between 9:00 and 11:00 a.m. with a similar number from each group on each day for three consecutive days. The time after the last exercise for sample collections were 27–29, 32.5–34.5, and 22.5–24.5 h, for ZT4, ZT12, and ZT22, respectively.

Blood samples were collected via cardiac puncture and centrifuged at 1,500 g for 20 min at 4°C, and the serum was separated and stored at −80°C until analyzed.

The right femur of each rat was quickly removed and cleaned of adherent tissue with razor blade for later RNA isolation. Then bones were wrapped in aluminum foil and flash-frozen in liquid nitrogen and stored at −80°C (Pedersen et al., 2019). The right tibia and lumbar vertebra of each animal were collected and stored in 70% ethanol before being submitted to structural and mechanical evaluation.

### Body composition

Body composition (fat compared with lean mass) was measured according to manufacturer’s instruction with an EchoMRI-100 whole body composition analyzer (Echo Medical Systems, LLC) 3 days before rat were euthanized. The analyzer uses quantitative magnetic resonance theory to determine fat mass, lean mass, and free water in scanned specimens, and the method has been validated in mice (Johnson et al., 2009). Individual live rat was placed in the tubular holder in the EchoMRI system, which was then activated through a software program.

### Bone structural and mechanical evaluation with micro computed tomography (µCT) and reference point indentation (RPI) instrument

The right tibia was removed from 70% ethanol, cleaned of adherent tissue, and soaked overnight in phosphate-buffered saline. The anterior portion of the mid-diaphysis of tibia was assessed using RPI (Biodent Hfc, Active Life Scientific, Santa Barbara, CA) according to the published method (Diez-Perez et al., 2010). Briefly, the reference probe, which housed a BP2 test probe, was lowered vertically to the surface, until it rested on the surface of the bone. In order to stabilize the unit, a reference force determined by the software was applied before each measurement was initiated. Each test included a series of 20 cycles at 2 Hz to a force of 5N. Bones were maintained in a hydrated state in phosphate buffered saline throughout the test. Five locations per sample, each ∼1–2 mm apart, were indented. Prior to testing, probes were tested on a Poly (methyl methacrylate) block according to manufacturers’ suggestion to ensure proper function. Total indentation distance (TID, µm) which is inversely correlated with toughness, and average energy dissipated (Avg ED, µJ), a measure of plasticity, were reported.

Then, the right tibia and the 2nd lumbar vertebra (L2) (Mathavan et al., 2015) from each rat were scanned for bone structure evaluation using a Scanco µCT scanner (µCT-40; Scanco Medical AG, Bassersdorf, Switzerland) at 16 µm isotropic voxel size with X-ray source power of 55 kV and 145 µA and integration time of 300 milliseconds. The L2 is The grey-scale images were processed using a low-pass Gaussian filter (sigma = 0.8, support = 1) to remove noise, and a fixed threshold of 275 was used to extract the mineralized bone from soft tissue and marrow phase. The reconstruction and 3D quantitative analyses were performed by using software provided by Scanco as previously described in detail (Cao et al., 2015). For femoral trabecular bone, the region of interest (ROI) consisted of 100 slices starting from about 1 mm distal to growth plate and for the L2 vertebra, the entire secondary spongiosa between the cranial and the caudal area were chosen for analyses. For assessment of cortical indexes, 100 slices at the tibial mid-diaphysis were scanned and analyzed. The following 3D parameters in the defined ROI were analyzed: bone volume (BV, mm^3^), tissue (cortical and marrow) volume (TV, mm^3^), relative bone volume over total volume (BV/TV, %), trabecular number (Tb.N, 1/mm), trabecular thickness (Tb.Th, μm), trabecular separation (Tb.Sp, μm), connectivity density (Conn.Dn, 1/mm3), structure model index (SMI, ranges from 0 to 3 with 0 = platelike and 3 = rodlike), and BMD (g hydroxyapatite/cm3). BMD is the average density of the segmented fraction of the ROI not including the marrow cavity. A calcium hydroxyapatite (HA) phantom (from 0 to 1,000 mg/cm^3^) is used for calibration The recommended guidelines for µCT scanning is followed (Bouxsein et al., 2010).

### Measurements of serum biochemical markers

The following bone-related serum cytokines were measured: tartrate-resistant acid phosphatase 5b (TRAP), propeptide of type 1 procollagen (P1NP), insulin-like growth factor-1 (IGF-1), and irisin. Exercise has been shown to increase serum IGF-1 (Yeh et al., 1994), which can stimulate bone formation, and irisin, a muscle secreted cytokine (Loffler et al., 2015). Irisin prevents bone loss in muscle disuse (Colaianni et al., 2017). Serum concentrations of bone biochemical markers were determined by using commercial anti-rat enzyme-linked immunosorbent assay kits according to the manufacturers’ instructions. Kits of tartrate-resistant acid phosphatase 5b (TRAP), CTX, and propeptide of type 1 procollagen (P1NP) were purchased from Immunodiagnostic System (Fountain Hill, AZ). Insulin-like growth factor-1 (IGF-1) and Irisin kits were purchased from R&D Systems (Minneapolis, MN) and G-Biosciences (St. Louis, MO), respectively. The same cytokine was measured for all samples in one batch preparation on the same day to minimize variation.

### Measurement of mRNA in whole bone

The frozen femur was pulverized with a liquid nitrogen-cooled steel mortar and pestle and total RNA was purified using Trizol (Carlsbad, CA) reagent according to the manufacturer’s instructions. Denatured total RNA from cells (2 µg) was reverse transcribed and amplified and quantified using a Sequence Detection System (SDS 7500) as previously described in detail (Cao et al., 2015). Relative mRNA expression was normalized to the expression of *Gapdh* mRNA in the same sample and delta delta CT method was used to calculate the fold changes of gene of interest relative to the No-Ex group. All oligonucleotide primers for PCR amplification were designed using PrimerQuest software, synthesized, and purified by HPLC by Integrated DNA Technologies (IDT, Coralville, IA).

The sense and antisense primer sequences were as follows: for Bmal1 5′-AGC TTA AGG TTT CAA ATG TGG AC-3′, and 5′-TGC TAC ATA TGC CAG TCT GAC-3’; for Clock 5′-TGC TAG AAA GAT GGA CAA GTC TAC-3′, and 5′-GGT TTC CAG TCC TGT CGA AT-3’; for Cry2 5′-GCA AGG AGG AGA GAC AGA AG-3′, and 5′-ATT GGC ATT CAT CCG AGG TC-3’; for Per2 5′-TCC TAG AAT TCC TCC CGA GAA G-3′, and 5′-TCA GAT CCT GAG GTA GAT AGC C-3’; for *Gapdh* 5′-ACA AGA TGG TGA AGG TCG GTG TGA-3′, and 5′-AGC TTC CCA TTC TCA GCC TTG ACT-3’. All oligonucleotide primers for PCR amplification were designed using PrimerQuest software and synthesized by Integrated DNA Technologies (IDT, Coralville, IA) with HPLC purification.

### Statistical analyses

Data are expressed as the mean ± SD. To test for differences between groups, data were analyzed using one-way ANOVA (JMP, version 15.0.0, SAS Institute, Inc.) followed by Tukey-Kramer *post hoc* contrasts among four groups if the main effect was significant. Planned comparison (*a priori* contrast) was performed to test whether the mean of the three Ex groups differed from the No-Ex group. The assumption of equal variance among groups was tested using Levene’s test and normality of data was check visually and with Shapiro-Wilk test using JMP. For all analyses, alpha was *a priori* set at *p* ≤ 0.05.

## Results

**FIGURE 2:**
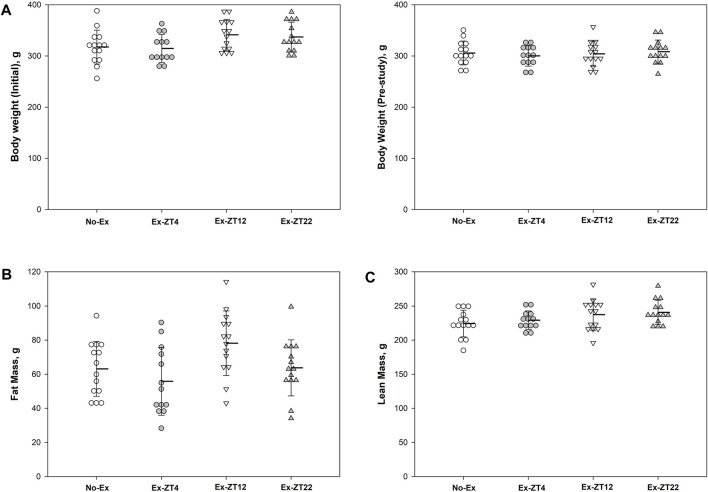
Initial **(A)** and final body weight **(B)**, fat mass **(C)**, lean mass **(D)**, and food intake **(E)** of 12-mo-old female Sprague Dawley rats exercised (Ex) at different Zeitgeber time (ZT) for 15 wk. Food intake was recorded for two consecutive days every two wk and the average for the study duration is presented. Data are the mean ± SD (*n* = 14/group, except n = 13 for Ex-ZT4). Data were analyzed using one-way ANOVA followed by Tukey-Kramer *post hoc* contrasts (JMP, version 15.0.0, SAS Institute, Inc.). The pooled Ex data were compared to the No-Ex data (*a priori* contrast). ZT0 is defined as light on, the start of the rest phase. ZT4, ZT12, ZT22, rats started involuntary exercise from the indicated ZT for 2 h.

The time of day of exercise did not affect body mass (Figure 2A). There were no differences in body mass of rats at end of the study among groups or the difference in body mass between at the end and the beginning of the study (15.8 ± 32.8, 14.3 ± 24.3, 35.6 ± 17.6, and 28.4 ± 18.3, for No-Ex, Ex-ZT4, Ex-ZT12, and Ex-ZT22, respectively, *p* = 0.07). There were no differences in fat mass, lean mass, or food intake among four groups (*p* = 0.07, 0.17, and 0.10, for fat mass, lean mass, and food intake, respectively) or between No-Ex and three Ex (Figures 2B, C, *p* = 0.86, 0.10, and 0.98, for fat mass, lean mass, and food intake, respectively).

**FIGURE 3:**
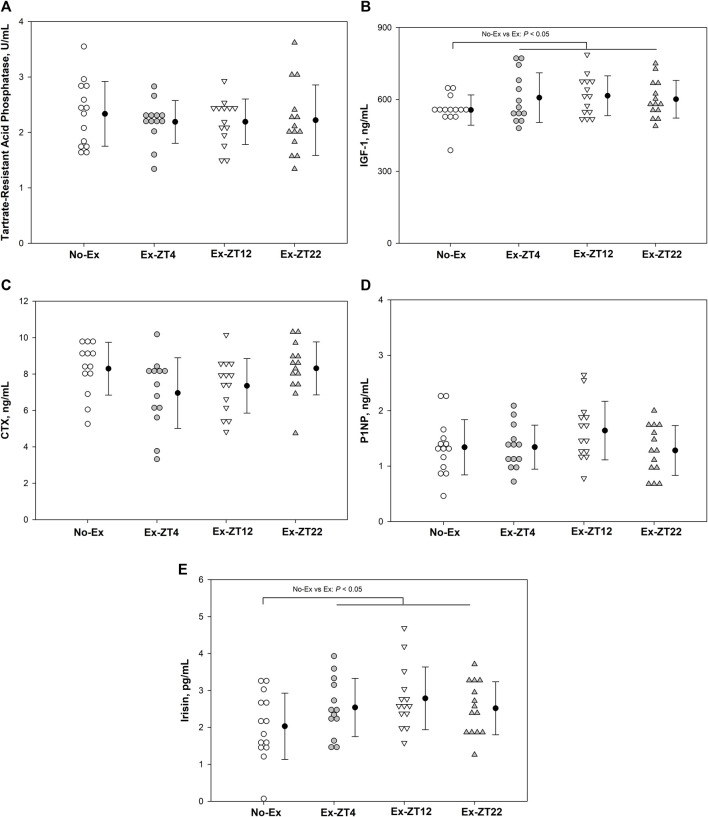
Serum concentrations of Tartrate-resistant acid phosphatase **(A)**, IGF-1 **(B)**, CTX **(C)**, P1NP **(D)**, and Irisin **(E)** in 12-mo-old female Sprague Dawley rats exercised (Ex) at different Zeitgeber time (ZT) for 15 wk. Data are the mean ± SD (*n* = 14/group, except *n* = 13 for Ex-ZT4). Data were analyzed using one-way ANOVA followed by Tukey-Kramer post hoc contrasts (JMP, version 15.0.0, SAS Institute, Inc.). Labeled means without a common letter differ, *P* < 0.05. The pooled Ex data were compared to the No-Ex data (*a priori* contrast). ZT0 is defined as light on, the start of the rest phase. ZT4, ZT12, ZT22, rats started involuntary exercise from the indicated ZT for 2 h.

Serum concentrations of IGF-1, TRAP, CTX, P1NP, or irisin were not affected the time of day of exercise (Figure 3, *p* = 0.21, 0.87, 0.08, 0.19, and 0.11, for IGF-1, TRAP, CTX, P1NP, or irisin, respectively). However, Rats in Ex groups had 10% and 30% greater serum concentrations of IGF-1 (Figure 3B, *p* = 0.03) and Irisin (Figure 3E, *p* = 0.02), respectively, than those of the No-Ex group.

**FIGURE 4:**
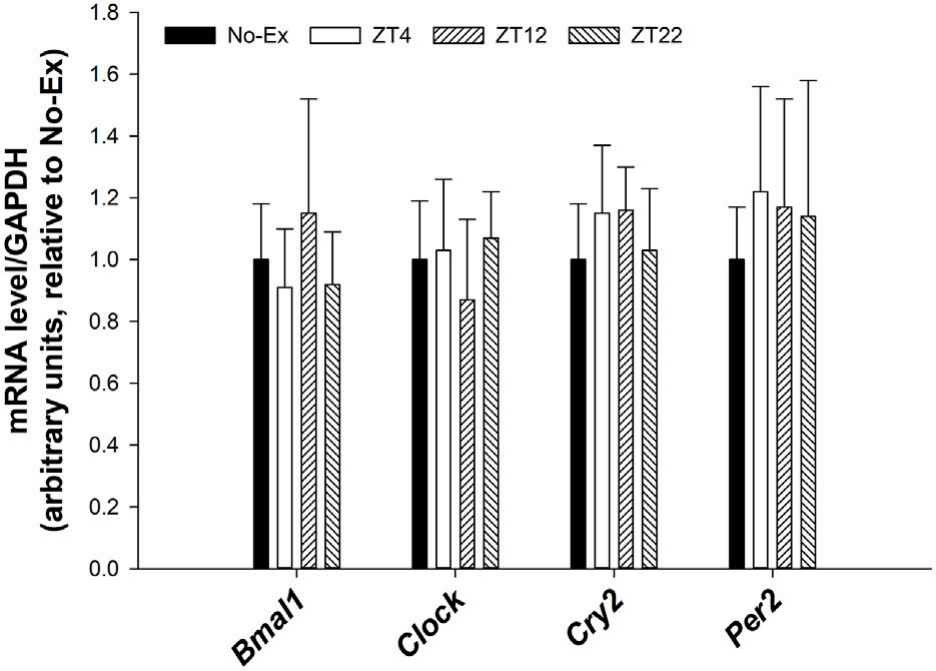
Expression of *Bmal1*, *Clock*, *Cry2*, and *Per2* in femur in 12-month-old female Sprague Dawley rats exercised (Ex) at different Zeitgeber time (ZT) for 15 weeks. Data are the mean ± SD (*n* = 14/group, except *n* = 13 for Ex-ZT4). Data were analyzed using one-way ANOVA followed by Tukey-Kramer *post hoc* contrasts (JMP, version 15.0.0, SAS Institute, Inc.). Labeled means without a common letter differ, *p* < 0.05. The pooled Ex data were compared to the No-Ex data (*a priori* contrast). ZT0 is defined as light on, the start of the rest phase. ZT (4, 12, 22), rats started involuntary exercise from the indicated ZT for 2 h.

There were no differences in expression of circadian genes such as *Bmal1*, *Clock*, *Cry2*, *Per2* in femur between either pooled Ex groups and No-Ex or among Ex groups at different ZT (Figure 4, *p* > 0.05).

Bone structure was scanned and analyzed by non-destructive μCT to evaluate the extent to which the time of day of Ex affected bone structure at distal and mid-diaphysis of the tibia and L2 (Table 1). Ex at different ZT did not affect bone structure parameters at tibial and L2 and Ex overall improved bone structure. For tibial bone structure, rats of pooled Ex groups had greater trabecular BV/TV by 16.7% (*p* = 0.01), Conn.Dn by 21.6% (*p* = 0.04), and BMD by 16.9% (*p* = 0.03), and lower SMI by 14.5% (*p* = 0.02), compared with No-Ex rats. For bone structure at L2, pooled Ex rats had 9.3% greater (*p* ≤ 0.05) Tb.Th compared to the No exercise group (*p* = 0.01). For tibial mid-diaphysis, Ex (pooled) decreased Me.Ar and increased Ct.Th by 38.1% and 7.7%, (*p* = 0.02 and 0.01, respectively). However, no differences in mechanical properties (total indentation distance or average energy dissipated) of tibial mid-diaphysis were detected between No-Ex and pooled Ex.

**TABLE 1:** Bone structural and mechanical properties of tibia and lumbar vertebrae in 12-month-old female Sprague Dawley rats exercised (Ex) at different Zeitgeber time (ZT) for 15 weeks^a^.

	No-Ex (*n* = 14)	Ex-ZT4 (*n* = 13)	Ex-ZT12 (*n* = 14)	Ex-ZT22 (*n* = 14)	ANOVA *p*-value	*A priori* contrast (No-Ex vs. pooled Ex) *p*-value
Proximal tibia (trabecular bone)						
TV, mm^3^	19.7 ± 1.9	20.1 ± 1.8	20.2 ± 2.4	19.8 ± 1.9	0.691	0.557
BV, mm^3^	3.52 ± 0.72	4.23 ± 0.65	4.17 ± 1.02	4.03 ± 0.87	0.319	0.132
BV/TV, %	17.8 ± 3.2	21.0 ± 2.3	20.6 ± 4.3	20.7 ± 3.2	0.052	0.006
Conn.Dn, mm^−3^	29.2 ± 9.8	37.1 ± 8.6	34.7 ± 7.6	34.8 ± 9.8	0.127	0.041
SMI	1.47 ± 0.33	0.94 ± 0.39	1.17 ± 0.39	1.19 ± 0.43	0.074	0.016
Tb.N, mm^−1^	2.39 ± 0.46	2.76 ± 0.38	2.64 ± 0.40	2.54 ± 0.26	0.275	0.143
Tb.Th, mm	0.09 ± 0.01	0.09 ± 0.01	0.09 ± 0.01	0.09 ± 0.01	0.399	0.218
Tb.Sp, mm	0.43 ± 0.09	0.36 ± 0.06	0.38 ± 0.07	0.39 ± 0.04	0.375	0.164
BMD, mg HA/cm^3^	189 ± 35	231 ± 26	219 ± 47	212 ± 40	0.115	0.032
Tibial mid-diaphysis (cortical bone)						
T.Ar, mm^2^	6.51 ± 0.38	6.38 ± 0.37	6.40 ± 0.39	6.49 ± 0.48	0.792	0.507
B.Ar, mm^2^	4.28 ± 0.30	4.48 ± 0.23	4.40 ± 0.26	4.42 ± 0.30	0.300	0.082
Me.Ar, mm^2^	2.23 ± 0.22	1.90 ± 0.34	2.00 ± 0.41	2.07 ± 0.29	0.073	0.023
Ct.Th, mm	0.52 ± 0.02^b^	0.57 ± 0.05^a^	0.55 ± 0.06^ab^	0.56 ± 0.03^ab^	0.038	0.006
TID, µm	53.3 ± 7.2	53.3 ± 7.0	55.0 ± 9.7	55.3 ± 11.0	0.893	0.658
Avg. ED, µJ	13.2 ± 3.0	12.1 ± 1.2	13.1 ± 2.6	12.5 ± 1.8	0.587	0.406
2nd Lumbar vertebrae (trabecular bone)						
TV, mm^3^	19.1 ± 2.0	18.6 ± 2.2	18.0 ± 3.6	19.5 ± 2.3	0.497	0.678
BV, mm^3^	6.49 ± 0.82	7.10 ± 1.63	6.68 ± 1.59	7.17 ± 1.01	0.464	0.230
BV/TV, %	34.2 ± 4.1	37.9 ± 6.3	37.2 ± 5.9	37.0 ± 4.7	0.294	0.061
Conn.Dn, mm^−3^	30.3 ± 3.4	26.0 ± 9.1	25.7 ± 6.8	28.5 ± 9.0	0.326	0.129
SMI	−0.34 ± 0.52	−0.72 ± 0.66	−0.71 ± 0.57	−0.51 ± 0.57	0.279	0.097
Tb.N, mm^−1^	3.05 ± 0.28	3.14 ± 0.42	3.08 ± 0.30	3.09 ± 0.31	0.898	0.574
Tb.Th, mm	0.11 ± 0.01	0.12 ± 0.02	0.12 ± 0.02	0.12 ± 0.01	0.093	0.012
Tb.Sp, mm	0.30 ± 0.03	0.30 ± 0.06	0.30 ± 0.04	0.30 ± 0.04	0.991	0.826
BMD, mg HA/cm^3^	364 ± 42	390 ± 51	379 ± 60	201 ± 14	0.565	0.191

^a^ Values are the mean ± SD., Data were analyzed using one-way ANOVA (JMP, version 15.0.0, SAS, institute, Inc.) followed by Tukey-Kramer *post hoc* contrasts. Labeled means in a row without a common letter differ, *p* < 0.05. The pooled Ex data were compared to the No-Ex data (*a priori* contrast). AvgED, average energy dissipated; B.Ar, cross-sectional bone area; BMD, bone mineral density; BV, bone volume; Conn.Dn, connectivity density; Ct.Th, cortical thickness; Me.Ar, cross-sectional medullary area; SMI, structure model index; T.Ar, cross-sectional total area; Tb.N, trabecular number; Tb.Sp, trabecular separation; Tb.Th, trabecular thickness; TID, total indentation distances; TV, total volume; ZT, zeitgeber time, ZT0 is defined as light on, the start of the rest phase. ZT (4, 12, 22), rats started involuntary exercise from the indicated ZT, for 2 h.

## Discussion

The main objective of this study was to investigate whether Ex-induced anabolic bone effect would differ when animals exercised at different ZT times to provide scientific basis for further improving bone structure with exercise. To our knowledge, this is the first study that assessed the time of day of Ex on bone-related changes in an old rat model. The major findings of this study with older female rats are as follows: 1) the time of day of Ex did not significantly affect bone structure and strength or serum bone biomarkers; 2) Ex improved tibial bone microarchitecture by increasing tibial bone mass, connectivity density, and decreasing structural model index; 3) Ex for 15 weeks did not affect body weight, fat mass, lean mass, or expression of circadian genes in bone.

In addition to compare active phase exercise to rest phase exercise, we decided to compare late vs. early phase exercise because studies showed that plasma concentration of bone resorption marker, C-terminal telopeptide of type 1 collagen (CTX), is the greatest in the early morning (early active phase) and the lowest in the afternoon (Bjarnason et al., 2002). In humans, exercise before breakfast increases fat oxidation over the next 24 h in men, an effect that does not occur when exercise is performed in the afternoon or evening (Iwayama et al., 2015). Ex, regardless of the mode of exercise, has many health benefits (Ruegsegger and Booth, 2018). For bone health, Ex is the most effective way to improve bone mass and strength by stimulating bone formation through mechanical loading (Turner and Robling, 2004; Warden et al., 2007; Ozcivici et al., 2010). Osteocytes, which account for more than 90% of cells in bone, in addition to osteoblasts and osteoclasts (Prideaux et al., 2016), sense mechanical loading induced by Ex. Ex increases bone mass by decreasing osteocyte secretion of sclerostin, a bone formation inhibiting protein (Armamento-Villareal et al., 2012; Macias et al., 2012).

The findings that Ex improved trabecular and cortical bone indices at the tibia but not at the lumbar spine in the current study are not surprising and indicate that the tibia, a weight-bearing long bone that undergoes many strain cycles during running exercise, is more sensitive to exercise than lumbar spine. Our results are consistent with those reported by Iwamoto et al. that moderate running exercise increases weight-bearing long bone but not lumbar vertebral bone mass (Iwamoto et al., 1998). Previously, with a similar number (13–14 in each group) of animals to this study, we demonstrated that Ex for 12 weeks decreased fat mass and serum concentrations of tartrate-resistant acid phosphatase (bone resorption marker), and improved bone structural properties of the distal femur in young rats (Cao et al., 2015; Cao, 2018). Older (12-month-old) female rats were used as a model to mimic bone deterioration with age and/or low estrogen in female individuals. However, we did not detect any differences in either body weight, fat mass, lean mass, the serum bone biomarkers, and bone structural and mechanical parameters between No-Ex and any individual Ex group. It appears that the effects of the same amount of Ex on bone-related endpoint outcomes were less apparent in older (12-month-old) female rats in this study as compared to the two previous studies with young rats. It is well established that bone’s adaptive response to mechanical stimuli is reduced with age due to intrinsic changes in aged animals such as aging-related changes in hormone status and cell function (Javaheri and Pitsillides, 2019). Therefore, it is likely the increased amount of Ex is needed in old animals to achieve detectable bone structural differences with the same number of animals. We found that Ex increased serum concentration of IGF-1, a bone anabolic agent that stimulates osteoblast proliferation and reduces osteoblast apoptosis (Cao et al., 2007), a finding that has been well established (Yeh et al., 1994; Franco et al., 2014). However, the serum IGF-1 concentration was lower in older female rats (∼600 ng/mL) in this study than those young rats used in our previous studies (∼1,000 mg/mL) (Cao et al., 2015; Cao, 2018). That the responsiveness of tissue, including bone, bone marrow stromal cells, or chondrocytes to IGF-1 decreases with aging (Martin et al., 1997; Cao et al., 2007) could be another contributing factor to the no detectable differences in bone-related outcomes between Ex and No-Ex groups, as compared to previous studies (Martin et al., 1997; Cao et al., 2007). Whether the time of day of Ex affects bone-related outcomes in young growing animals remains to be investigated. Ex may affect bone turnover through muscle secreted signaling factors as seen in this study that Ex increased serum irisin concentration. Similarly, short bouts of intensive exercise transiently increase serum irisin levels in children and adults (Loffler et al., 2015) and administration of irisin prevents bone loss and muscle atrophy in hind-limb suspended mice (Colaianni et al., 2017).

Indeed, bone resorption and formation markers exhibit diurnal patterns with light/dark cycle in nocturnal rats (Shao et al., 2003) or mice (Srivastava et al., 2001) that peaked during the light period when animals are in rest phase and the patterns are opposite to those observed in humans according to clock time (Bjarnason et al., 2002; Redmond et al., 2016; Swanson C. et al., 2017; Swanson et al., 2017c). Although physical activities have a positive effect on bone in general, different exercise models (treadmill running, wheel running, swimming, resistance training, and vibration modes) may have different osteogenic effects (Portier et al., 2020). The finding that the time of day of Ex did not affect bone structural or mechanical parameters in this study is somewhat unexpected. We hypothesized that Ex in the rest phase (light on) would disrupt animals’ normal circadian rhythm and blunt Ex-induced bone anabolic effect, and that Ex in active phase (light off) would be superior to exercise in rest phase in improving bone-related outcomes in older female rats. While rest phase Ex might disrupt animals’ circadian rhythms which have negative impact on bone, Ex during the rest phase could have greater suppression of bone resorption, which is high during the rest phase, compared to Ex during the light phase. Rogers et al. reported that Ex acutely decreases serum concentrations of bone resorption markers, such as TRAP5b and PTH (Rogers et al., 2011). Limited evidence suggests young growing animals may be more sensitive to the time of day of Ex than old animals used in this study, as discussed above. Bouchard et al. reported that *in vivo* cyclic compressive loading at ZT2 (resting phase) resulted in upregulation of *Sost*, bone formation inhibitor, and loading at ZT14 (early active phase) resulted in a greater bone formation response and decreased *Sost* and *Dkk1* expression compared to 10-week old female mice loaded at ZT2 (Bouchard et al., 2022).

In this study, the time of day of Ex did not affect bone biomarkers or expression of circadian genes. Similarly, Bouchard et al. reported that *in vivo* cyclic compressive loading did not affect the expression of clock genes (Bmal1, Clock, Per1, and Per2) and the authors suggest that the bone’s innate circadian rhythm is unaffected by loading (Bouchard et al., 2022). However, it has been reported that Ex upregulated circadian clock genes, such as Cry1, Per1, and Bmal1, in human skeletal muscle 6 h after resistance Ex (Zambon et al., 2003) and Ex also induced phase-shifted effects on circadian clocks in muscle (Aoyama and Shibata, 2017). Ex may have little influence on circadian rhythm of bone biomarkers. For example, Kim et al. (2000) showed that unloading for 14 days affected bone resorption markers but did not disrupt the basic bone resorption diurnal rhythm. Pedersen et al. (1995) also reported that bed rest did not change the circadian rhythm of serum CTX, alkaline phosphatase, or osteocalcin in humans. Therefore, the timing of the sample collections could contribute to the lack of difference in bone biomarkers or expression of circadian genes among the time of day of Ex groups. In this study, animals were sacrificed, and samples were collected between 9:00 and 11:00a.m. which corresponded to ZT3 for No-Ex and Ex-ZT4 groups, and ZT8.5 and ZT23 for Ex-ZT12 and Ex-ZT22 based on light schedule for each group, respectively. The importance of the time of day for sample collection has been discussed (Nelson et al., 2022). Normally samples should be collected at the same ZT time to minimize variability due to circadian rhythms. Considering that Ex also acutely affects bone biomarkers (Rogers et al., 2011; Gombos et al., 2016), the time of sample collection after Ex could be a further confounding factor for interpretation of any differences in serum bone biomarkers. Calcium intake and excretion were not measured in this study. The potential interplay among circadian rhythm, calcium metabolism, and exercise need be further investigated.

Although both animals and humans exhibit biological rhythms (Aoyama and Shibata, 2017), extrapolating data from nocturnal rodents to diurnal humans should be taken with caution. Few environmental cues affecting the biological rhythms and the responses to these environmental changes could be distinctively different for animals living in vivarium as compared to humans. For example, animals live in a controlled vivarium with the identical lighting intensity (normally 12 h light/dark cycle), humidity, whereas humans experience varying light intensity, humidity throughout the day, season, or year. Biological rhythms in humans are also sensitive to the changes in eating pattern and diet composition (Bjarnason et al., 2002) whereas animals have constant access to the same diet. Animals during rest phase usually experience greater environmental sound (or noise) than active phase while humans are opposite. Therefore, findings in this animal study do not suggest the time of day of exercise would not affect bone metabolisms in humans.

In summary, our data demonstrate that exercise is beneficial to bone health regardless of the time of day of exercise in older female rats.

## Data Availability

The original contributions presented in the study are included in the article/Supplementary Material, further inquiries can be directed to the corresponding author.

## Abbreviations

µCT, micro computed tomography; AvgED, average energy dissipated; B.Ar, bone area; BMD, bone mineral density; BV, bone volume; Conn.D, connectivity density; Ct.Th, cortical thickness; CTX, C-terminal telopeptide of type 1 collagen; IGF-1, insulin-like growth factor; L2, 2nd lumbar vertebra; Me.Ar, medullary area; P1NP, propeptide of type 1 procollagen; SMI, structure model index; T.Ar, total area; Tb.N, trabecular number; Tb.Sp, trabecular separation; Tb.Th, trabecular thickness; TID, total indentation distances; TRAP, tartrate-resistant acid phosphatase; TV, total volume; ZT, Zeitgeber time, ZT 0 is defined as light on, the start of the rest phase.
